# Clinical efficacy of minimally invasive transforaminal lumbar interbody fusion (MIS-TLIF) in the treatment of II° lumbar isthmic spondylolisthesis: A retrospective cohort study

**DOI:** 10.1097/MD.0000000000035420

**Published:** 2023-10-06

**Authors:** Bin Zhang, Jun-Song Ma, Pin Feng, Yuan Hu, Jun-Lin Liu, Qing-Quan Kong

**Affiliations:** a Department of Orthopedics Surgery, Hospital of Chengdu Office of People’s Government of Tibetan Autonomous Region, Chengdu, Sichuan, China; b Department of Orthopedics Surgery, West China Hospital, Sichuan University, Chengdu, Sichuan, China.

**Keywords:** lumbar fusion, lumbar isthmic spondylolisthesis, minimally invasive surgery

## Abstract

Minimally invasive transforaminal lumbar interbody fusion (MIS-TLIF) is not suitable for high-grade isthmic spondylolisthesis, whether MIS-TLIF can treat II° lumbar isthmic spondylolisthesis (IS) is still controversial. This retrospective cohort study compared the clinical efficacy of MIS-TLIF and open transforaminal lumbar interbody fusion (OPEN-TLIF) in the treatment of II° lumbar IS. From January 2017 to January 2023, 101 patients with II° lumbar IS were diagnosed in our hospital and underwent surgical treatment, of which 53 received MIS-TLIF surgery and 48 received OPEN-TLIF surgery. The operation time, blood loss and surgical complications were compared between the 2 groups. The pain, function, reduction rate and fusion rate of the patients were evaluated during follow-up. The amount of intraoperative blood loss, postoperative drainage, and postoperative hospital stay in the MIS-TLIF group were significantly lower than those in the OPEN-TLIF group were (*P* < .01). In the MIS-TLIF group, there were 1 case of dural sac injury and 3 cases of lower limb paralysis. The complication rate of MIS-TLIF was lower than the OPEN-TLIF group (*P* = .032). In the visual analog scale score of low back pain, the MIS-TLIF group was lower than the OPEN-TLIF group after operation and at the last follow-up. There were no significant differences in postoperative leg pain score, slippage rate, and fusion rate between the 2 groups. Compared with OPEN-TLIF, MIS-TLIF has the advantages of better low back pain relief, less trauma, less bleeding and faster recovery, and is worthy of clinical promotion.

## 1. Introduction

Lumbar spondylolysis is a bone discontinuity or defect caused by congenital or acquired fatigue fractures of the isthmus, most commonly in the lumbar 4 and 5 vertebrae. Isthmic spondylolisthesis (IS) is a common type of lumbar spondylolisthesis, especially in young and middle-aged people.^[1–4]^ The clinical symptoms are low back pain after activity and exertion, often accompanied by radicular pain in the lower extremities. Symptomatic lumbar isthmic spondylolisthesis often requires surgical treatment after conservative treatment fails.^[5–9]^

Lumbar fusion is the standard surgical approach for the treatment of IS.^[7,9,10]^ The traditional open transforaminal lumbar interbody fusion (OPEN-TLIF) is suitable for all types of IS. OPEN-TLIF requires extensive stripping of paraspinal muscles and soft tissues, which has the disadvantages of excessive bleeding and long recovery time.^[7,10–12]^ Minimally invasive transforaminal lumbar interbody fusion (MIS-TLIF) is performed through the intermuscular approach, which causes less damage to the paraspinal muscles and preserves the posterior ligament complex. It has the advantages of less trauma, less bleeding, and faster recovery. MIS-TLIF is suitable for I° IS,^[13]^ but its effect on spondylolisthesis reduction is limited, and it is not suitable for high-grade spondylolisthesis.^[14,15]^ Whether MIS-TLIF is suitable for II° spondylolisthesis is still controversial.^[4,16–19]^ Because II° spondylolisthesis accounts for the highest proportion of lumbar IS that requires surgical treatment, this retrospective cohort study compared the clinical efficacy and complications of OPEN-TLIF and MIS-TLIF in the treatment of lumbar single-level II°IS.

## 2. Materials and methods

### 2.1. Patient selection

This study was approved by the institutional review board (#202038) and performed in accordance with the Declaration of Helsinki. We retrospectively reviewed 136 consecutive patients with single-level II° IS at our hospital, who underwent surgery between January 2017 and January 2023. The patients were screened according to the following inclusion and exclusion criteria.

#### 1.2.1. Inclusion criteria

1. Single-segment bilateral lumbar spondylolysis was confirmed by preoperative imaging examination, and the spondylolisthesis was Meyerding II°, that is, the vertebral body slipped forward by 25% to 50%.
2. Low back pain with or without radiculopathy.
3. Patients who failed conservative treatment for more than 3 months or whose symptoms significantly affected their daily life.
4. The surgical method was open or minimally invasive transforaminal lumbar intervertebral fusion.

#### 2.2.1. Exclusion criteria

1. Patients with lumbar multi-segmental lesions requiring surgical treatment.
2. Spine tumors, infections, severe scoliosis, etc.
3. Patients with incomplete follow-up data.

According to the above inclusion and exclusion criteria, we collected 101 patients with complete data, of which 48 underwent OPEN-TLIF surgery and 53 underwent MIS-TLIF surgery. The baseline data of the 2 groups were shown in Table 1. There were no statistical differences in gender, age, body mass index, and surgical segment between the 2 groups.

**Table 1:** Comparison of baseline data of two groups.

	OPEN-TLIF (n = 48)	MIS-TLIF (n = 53)	*P* value
Sex (M/F)	25/23	29/16	Z = −0.303; *P* = .762
Age (y)	59.96 ± 9.96	58.94 ± 10.43	T = 0.567; *P* = .571
BMI (kg/m^2^)	25.50 ± 1.17	25.51 ± 1.43	T = −0.031; *P* = .975
Lumbar levels			Z = −0.366; *P* = .714
L3	9	10	
L4	18	22	
L5	21	21	
Follow-up time (mo)	18.54 ± 6.31	18.96 ± 5.88	T = −0.402; *P* = .688

BMI = body mass index, MIS-TLIF = minimally invasive transforaminal lumbar interbody fusion, OPEN-TLIF = open transforaminal lumbar interbody fusion.

### 2.2. Surgical method

All patients in the 2 groups were under general anesthesia, lying prone on an adjustable lumbar bridge and radio-permeable table, abdominal suspension, and the operation segment was marked with C-arm fluoroscopy before operation. A drainage tube was placed before the wound was sutured in all operations, and the wound was sutured layer by layer. Antibiotics were used prophylactically for 24 hours. Lumbar X-ray and 3-dimensional CT were checked on the first day after operation, remove the drainage tube when the drainage volume was < 50 mL. Wear a lumbar support for activities within 3 months, and avoid bending, twisting, and weight bearing.

#### 2.2.1. OPEN-TLIF procedure

A longitudinal incision of about 10 cm was made in the middle of the waist. The skin, subcutaneous tissue, and deep fascia were incised layer by layer. The paraspinal muscles were stripped subperiosteally along both sides of the spinous process to fully expose the bilateral lamina, isthmus, articular process, and transverse process. Referring to the apex of the “herringbone crest” and the midpoint of the transverse process as the needle insertion point, 4 pedicle screws were implanted. Afterwards, the bilateral articular processes and lamina were resected with an ultrasonic osteotome combined with a lamina rongeur, and the scar tissue and ligamentum flavum in the isthmus were carefully removed to protect the nerve roots and the dural sac. Carefully identify the intervertebral space below the dural sac. After cutting the fibrous annulus, use a blade reamer to insert into the space in parallel. Gradually remove the nucleus pulposus tissue and cartilage endplate until the bony endplate was ready. After the intervertebral space was fully released, a large-sized fusion model was implanted step by step. Fluoroscopy was used to confirm the reduction of the spondylolisthesis. If the reduction was not satisfactory, the contralateral pedicle screw was pulled to assist the reduction after the intervertebral space was enlarged. After satisfactory reduction, a fusion cage was placed after intervertebral bone grafting. Appropriate pressure was applied after connecting rods, and horizontal connection was installed if necessary.

#### 2.2.2. MIS-TLIF procedure

The C-arm fluoroscopy was used to determine the responsible segment, and the projection of the upper and lower pedicles and the midline of the spinous process were marked on the body surface. The surgical incision was a longitudinal incision 1cm away from the midpoint of the upper and lower pedicles. After routine disinfection and draping, under C-arm fluoroscopy, 2 o’clock and 11 o’clock of the pedicle projection were selected as the needle insertion points. Under fluoroscopy, the puncture needle gradually passed through the pedicle and entered the vertebral body, and was replaced with a memory guide wire. After incision of the skin, subcutaneous and deep fascia, identify the muscle space, expand the muscle space step by step and place a 24/28 tapered channel, which fixed by free arm. The soft tissues were cleaned to expose isthmus, lamina and articular process. The facet joint was resected step by step with an ultrasonic osteotome, and hypertrophic osteophytes and scar tissue in the isthmus were removed. Expose and protect the dural sac and nerve roots. Identify the intervertebral space and incise the annulus fibrosus, insert a sheet-shaped reamer into the space in parallel to fully release the intervertebral space. Expose and resect the contralateral articular process and release the contralateral intervertebral space in the same way. Gradually remove the nucleus pulposus tissue and cartilage endplate until the bony endplate was ready. After the intervertebral space was fully released, a large-sized fusion test model was implanted step by step. Fluoroscopy was used to confirm the reduction of the spondylolisthesis. If the reduction was not satisfactory, the contralateral pedicle screws were used to expand the disc space and then pulled to assist reduction. After satisfactory reduction, a fusion cage was placed after intervertebral bone grafting. Finally, the intervertebral space is appropriately compressed by pedicle screws.

### 2.3. Evaluation indicators

The operation time, intraoperative blood loss and postoperative drainage volume, postoperative hospital stay, and operation-related complications were recorded. Low back pain and lower extremity pain were assessed using visual analogue scores (VAS).^[20]^ Oswestry disability index (ODI)^[21]^ was used to evaluate the functional recovery of patients. During the follow-up period, X-ray and sagittal CT were used to measure the reduction rate of spondylolisthesis and evaluate the intervertebral fusion.^[13,14]^

### 2.4. Statistical methods

SPSS 21.0 statistical software (IBM Corp., Armonk, NY) was used for analysis, and measurement data were expressed as mean ± standard deviation. The rank sum test was used to compare the baseline count data between the 2 groups, and the independent sample *t* test was used to compare the measurement data such as age, bleeding volume, postoperative hospital stay, VAS score, ODI score, and slippage rate between the 2 groups. *P* < .05 is considered statistically significant.

## 3. Results

### 3.1. Postoperative general situation

Both groups of patients completed the operation successfully. The patients in the OPEN-TLIF group were followed up for an average of 18.54 ± 6.31 months, and the patients in the MIS-TLIF group were followed up for an average of 18.96 ± 5.88 months. In the OPEN-TLIF group, the average operation time was 148.02 ± 19.67 minutes, the intraoperative blood loss was 284.58 ± 80.85 mL, the postoperative drainage volume was 158.33 ± 77.14 mL, and the average postoperative hospital stay was 6.19 ± 1.38 days. There were 2 cases of dural sac injury, 5 cases paralysis of lower extremities, and wound infection in 2 cases. The dural tear was repaired intraoperatively, and the deep fascia was tightly sutured, and the drainage tube was pulled out 3 days after the operation. Patients with lower limb paralysis improved after 1 to 2 months of neurotrophic drugs. The 2 cases of wound infection were both superficial infections, and they healed after intensive dressing changes.

In the MIS-TLIF group, the average operation time was 154.43 ± 20.04 minutes, intraoperative blood loss was 174.72 ± 47.23 mL, postoperative drainage volume was 51.13 ± 24.51 mL, postoperative hospital stay was 3.87 ± 0.98 days. There were 1 case of dural sac injury, 2 cases of lower extremity paralysis, no wound infection (Table 2). The dural sac injury was sutured during the operation, and no cerebrospinal fluid leakage was found after the operation. Patients with lower limb paralysis improved after being treated with neurotrophic drugs.

**Table 2:** Comparison of surgery-related data between the two groups.

	OPEN-TLIF	MIS-TLIF	*P* value
Operation time (min)	148.02 ± 19.67	154.43 ± 20.04	T = −1.850; *P* = .066
Bleeding (mL)			
Intraoperative blood loss	284.58 ± 80.85	174.72 ± 47.23	T = 10.581; *P* = .000
Postoperative drainage	158.33 ± 77.14	51.13 ± 24.51	T = 12.977; *P* = .000
Postoperative hospital stay	6.19 ± 1.38	3.87 ± 0.98	T = 11.942; *P* = .000
Complications			Z = −2.109; *P* = .032
Dural tear	2	1	
Nerve paralysis	5	3	
Wound infection	2	0	

MIS-TLIF = minimally invasive transforaminal lumbar interbody fusion, OPEN-TLIF = open transforaminal lumbar interbody fusion.

### 3.2. Pain and function evaluation

The low back VAS of the OPEN-TLIF group was 6.71 ± 1.27 before operation, 3.33 ± 0.95 on the second day after operation, and 1.81 ± 0.89 at last follow-up. The score of MIS-TLIF group was 6.42 ± 1.19 before operation, 2.79 ± 0.96 on the second day after operation, and 0.91 ± 0.63 at the last follow-up. The postoperative low back pain and ODI in the MIS-TLIF group were significantly lower than those in the OPEN-TLIF group during the follow-up. However, there was no significant difference in leg VAS score between the 2 groups after operation and at the last follow-up. The preoperative and postoperative low back pain VAS, leg pain VAS and ODI scores were shown in Table 3.

**Table 3:** Comparison of VAS and ODI data between the two groups.

	Open-TLIF	MIS-TLIF	*P* value
VAS of low back			
Preoperative	6.71 ± 1.27	6.42 ± 1.19	T = 1.383; *P* = .169
Postoperative 2nd d	3.33 ± 0.95	2.79 ± 0.96	T = 3.238; *P* = .001
Last follow-up	1.81 ± 0.89	0.91 ± 0.63	T = 7.259; *P* = .000
VAS of leg			
Preoperative	4.71 ± 1.54	4.98 ± 0.99	T = −1.322; *P* = .188
Postoperative 2nd d	1.73 ± 0.92	2.02 ± 0.88	T = −1.863; *P* = .064
Last follow-up	0.88 ± 0.61	0.83 ± 0.67	T = 0.396; *P* = .692
ODI			
Preoperative	60.93 ± 8.99	62.83 ± 7.90	T = −1.318; *P* = .189
Last follow-up	26.38 ± 7.67	24.00 ± 5.80	T = 2.120; *P* = .036

MIS-TLIF = minimally invasive transforaminal lumbar interbody fusion, ODI = oswestry disability index, OPEN-TLIF = open transforaminal lumbar interbody fusion, VAS = visual analog scale.

### 3.3. Imaging evaluation

**Figure 1.**
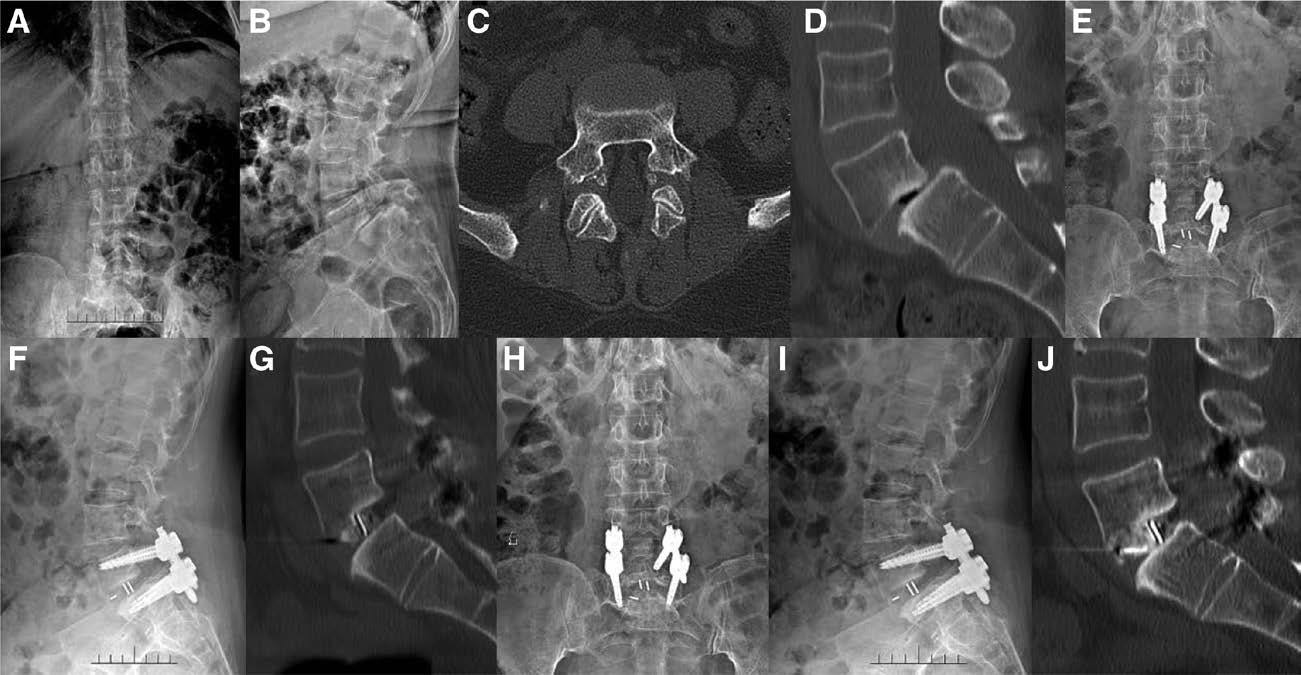
A 63-year-old female patient with bilateral isthmic spondylolisthesis (II°) of the L5 vertebra underwent MIS-TLIF. (A, B) Preoperative X-ray of the lumbar spine shows anterior spondylolisthesis of lumbar 5; (C, D) Preoperative lumbar CT shows bilateral isthmus of lumbar 5 vertebrae, with anterior spondylolisthesis II°; (E, F, G) The X-ray and CT examination 1 day after operation showed that the slippage was reduced satisfactorily and the internal fixation position was good. (H, I, J) X-ray and CT examination 1 year after operation showed good reduction of spondylolisthesis and intervertebral bony fusion. MIS-TLIF = minimally invasive transforaminal lumbar interbody fusion.

The lumbar spondylolisthesis rates of the 2 groups were significantly improved on the 1st day after operation and at the last follow-up compared with those before operation, but there was no statistical difference between the 2 groups (*P* < .05). At the last follow-up, the fusion rate was 95.83% (46/48) in the OPEN-TLIF group and 96.23% (51/53) in the MIS-TLIF group, and there was no statistical difference between the 2 groups (Table 4). The image data of typical case was shown in Figure 1.

**Table 4:** Comparison of slip percentage and fusion rate between the two groups.

	OPEN-TLIF	MIS-TLIF	*P* value
Slip percentage			
Preoperative	35.22 ± 5.18	36.40 ± 4.79	T = −1.374; *P* = .171
Postoperative 2nd d	7.65 ± 1.97	7.58 ± 1.91	T = 0.229; *P* = .819
Last follow-up	8.21 ± 2.00	8.29 ± 1.99	T = −0.230; *P* = .819
Fusion rate	46/48(95.83%)	51/53(96.23%)	Z = −0.116; *P* = .907

MIS-TLIF = minimally invasive transforaminal lumbar interbody fusion, OPEN-TLIF = open transforaminal lumbar interbody fusion.

## 4. Discussion

Lumbar IS is a common type of spondylolisthesis caused by congenital or acquired bone discontinuity in the isthmus, which leads to forward spondylolisthesis of the vertebral body.^[2–4]^ Lumbar isthmic spondylolisthesis often occurs in the lumbar 4 and 5. The Meyerding classification divides spondylolisthesis into 5 degrees: I°, forward spondylolisthesis is < 25% of the anterior-posterior diameter of the lower vertebra; II° spondylolisthesis is 25% to 50%; III° spondylolisthesis is 50% to 75%; IV° spondylolisthesis is 75% to 100%; V° spondylolisthesis is > 100%, complete slippage.^[4]^ Among them, I° and II° are low-grade spondylolisthesis, III° and above are high-grade spondylolisthesis. Asymptomatic mild isthmic spondylolisthesis generally does not require treatment. For symptomatic patients with isthmic spondylolisthesis, conservative treatment is the first choice. Conservative treatment is the first choice for patients with symptomatic isthmic spondylolisthesis.^[4–6]^ Surgical treatment is required for patients who have no obvious relief from conservative treatment and have symptoms of lower extremity nerves or cauda equina syndrome.^[6]^

The purpose of surgery is to relieve pain and improve function, nerve decompression, and reduction of spondylolisthesis and restoration of lumbar stability.^[4–7]^ For high-grade spondylolisthesis above III°, reduction, decompression and fusion are recommended, but there is no consensus on the surgical treatment of mild spondylolisthesis of Meyerding grade I to II.^[7,22]^ Surgical treatment of I°IS mainly includes isthmic repair and lumbar fusion.^[23,24]^ Isthmus repair requires removal of scars in the isthmus and bone grafting. Screws or other instruments are used to assist fixation in the isthmus to promote healing.^[25,26]^ Since the isthmus of the Asian population is narrower than that of the European and American population, the probability of bone graft non-fusion in the isthmus is higher, and this surgical procedure may lead to postoperative low back pain.^[27]^ The long-term follow-up of the researchers found that the quality of life scores of patients who underwent isthmus repair decreased over time, and the degeneration of the intervertebral disc at the responsible segment did not slow down.^[28]^ Therefore, isthmus repair is suitable for young spondylolisthesis patients without disc degeneration. For patients with II° IS, most of them are accompanied by degeneration and protrusion of intervertebral disc, scarring, osteophyte hyperplasia, resulting in compression of nerves. Surgery requires complete scar removal and reduction of spondylolisthesis to achieve the purpose of nerve decompression. Therefore, lumbar fusion is more advantageous for patients with II° IS who fail conservative treatment.^[7–9]^

For II° IS, the most common lumbar fusion procedures include open and minimally invasive TLIF procedures. The traditional OPEN-TLIF surgery requires extensive stripping of the bilateral paraspinal muscles to expose the bony structure. The surgical trauma is large and there is a lot of bleeding. At the same time, the denervation of the paraspinal muscles is an important cause of postoperative low back pain.^[29]^ MIS-TLIF has less surgical trauma, less interference to the paraspinal soft tissue through the intermuscular space, and has positive significance for maintaining spinal stability, and the amount of blood loss is lower than that of open surgery.

In this study, the intraoperative blood loss and postoperative drainage in the MIS-TLIF group were lower than those in the OPEN-TLIF group. There were 2 cases of postoperative wound complications in the OPEN-TLIF group, which was significantly higher than that in the MIS-TLIF group. The low back pain score after MIS-TLIF was significantly lower than that of the OPEN-TLIF group, which may be related to less damage to paravertebral muscles and soft tissues during the MIS-TLIF operation. At the same time, the postoperative hospital stay of the MIS-TLIF group was lower than that of the OPEN-TLIF group, indicating that MIS-TLIF has less trauma and can promote the recovery of patients faster.

In terms of surgical effect, both of the 2 surgical methods can better improve the patient’s symptoms and improve the quality of life. Our results found that the MIS-TLIF group showed better improvement in the low back pain VAS scores in the early and last fellow-up compared with the OPEN-TLIF group. These indicate that the MIS-TLIF technique only performs bilateral facetectomy, preserving most of the posterior anatomical structure of the lumbar spine, which can effectively avoid the extensive stripping of the paraspinal muscles and damage to the lamina, ligamentum flavum and articular process structures in the OPEN-TLIF surgery. The last follow-up results showed that the leg pain VAS scores in the MIS-TLIF group were similar to those in the OPEN-TLIF group, and both groups showed better clinical improvement. At the same time, there was no significant difference in the reduction of spondylolisthesis and fusion rate between the 2 groups. It shows that MIS-TLIF has less trauma, less bleeding, fewer complications, and faster recovery compared with OPEN-TLIF, which is in line with the current concept of enhanced rehabilitation surgery and is more conducive to the recovery of patients.

At present, there are still controversies on whether lumbar spondylolisthesis requires complete reduction, the indications for reduction, and the methods of reduction. However, effective reduction of spondylolisthesis, and adequate correction of deformity have become important indicators for evaluating the effect of surgery.^[7,30–32]^ The reduction of spondylolisthesis and the correction of deformity are significantly related to the clinical effect and the satisfaction rate of patients.^[33]^ Reduction will improve the global sagittal alignment and improve the biomechanical environment for fusion. In this study, the sagittal slip percentage in the MIS-TLIF group decreased from 36.40 ± 4.79 before operation to 7.58 ± 1.91 after operation, and was 8.29 ± 1.99 at the last follow-up, which was significantly improved compared with preoperatively. The fusion rate at the last follow-up was 96.23%, indicating that the MIS-TLIF technique can not only effectively reduce the spondylolisthesis, but also effectively correct the lumbar lordosis and maintain the spondylolisthesis reduction. These results were similar to those of the OPEN-TLIF group, indicating that even under the minimally invasive channel, hyperosteophytes and scars in the bilateral isthmus can still be effectively removed, the intervertebral space can be fully released, and spondylolisthesis can be reduced.

For lumbar II° IS, although some patients have no lower extremity symptoms, if the isthmic hypertrophic scar and osteophyte tissue are not decompressed, new compression on the dural sac and nerves, especially the exiting nerve root, may be caused after the spondylolisthesis is reduction. Bilateral decompression is therefore recommended for patients with isthmic spondylolisthesis.^[34,35]^ MIS-TLIF operation using bilateral surgical approach is an effective decompression and reduction method.^[13]^ The bilateral approach can not only achieve effective decompression of the bilateral isthmus and lateral recess, but also further treat and release the intervertebral space, and effectively assist the reduction of spondylolisthesis. In patients with lumbar isthmic spondylolisthesis, the dural sac and band-shaped scars around the nerves are severely adhered. During the operation, the inferior articular process should be resected first, and then the stump of the isthmus should be resected gradually toward the cephalad, and the hypertrophic scar and osteophyte tissue of the isthmus should be resected. The adhesion between the scar tissue and the dural sac was separated from the insertion point of the ligamentum flavum, and the ligamentum flavum was gradually removed. If the osteophyte tissue severely compresses the nerve during decompression of the isthmus, small lamina rongeurs can be used to gradually remove the osteophyte, or an ultrasonic osteotome can be used to remove the osteophyte in pieces. Pay attention to protecting the traveling nerve root below the osteophyte to avoid crushing and damaging the nerve root. When dealing with the intervertebral space, the direction of the channel should be adjusted to be parallel to the intervertebral space to avoid damage to the bony endplate. When performing spondylolisthesis reduction, the bilateral articular processes, osteophyte, and scar tissue should be removed first, and the bilateral intervertebral space should be fully released. If the reduction is still insufficient, the contralateral pedicle screws are used to expand the gap and then pulled to assist reduction and temporary fixation. After the reduction is satisfactory, a fusion cage is placed after the intervertebral bone graft. Finally, the intervertebral space is appropriately compressed by pedicle screws.

## 5. Limitations

Our study has some limitations. First, the number of patients enrolled in this study was small, and compared with large samples, small trials are more likely to overestimate the treatment effect. Second, this study mainly focused on the short-term parameters aimed at promoting accelerated postoperative recovery, and a longer follow-up time was needed to evaluate the long-term clinical outcomes between the MIS-TLIF and the OPEN-TLIF group. Third, all operations were performed by a single experienced surgeon, and results may vary if a surgeon with different experience performs the MIS-TLIF procedure. In the later stage, prospective randomized controlled trials with large samples and long-term follow-up are needed for further verification.

In summary, MIS-TLIF in the treatment of single-segment lumbar II° IS has the advantages of less trauma, quick recovery, and good clinical effect, and is worthy of clinical application.

## Author contributions

**Conceptualization:** Bin Zhang, Yuan Hu.

**Data curation:** Bin Zhang, Pin Feng.

**Funding acquisition:** Bin Zhang.

**Investigation:** Jun-Song Ma.

**Methodology:** Jun-Song Ma, Yuan Hu.

**Project administration:** Jun-Song Ma, Qingquan Kong.

**Software:** Jun-Song Ma.

**Validation:** Jun-Song Ma, Pin Feng.

**Visualization:** Pin Feng, Qingquan Kong.

**Writing – original draft:** Bin Zhang, Yuan Hu, Jun-Lin Liu.

**Writing – review & editing:** Qingquan Kong.
